# Deranged Uterine Function

**Published:** 1907-11

**Authors:** James A. Black

**Affiliations:** Morganza, Pa.


### Deranged Uterine Function.

JAMES A. BLACK, M. D., MORGANZA, PA.

It is safe to say that to the average physician, who is confronted almost daily with the ordinary cases of suppressed and deranged uterine function, no other class of cases is so uniformly disappointing in results and yields so sparing a return for the care and time devoted to their conduct.

Patients suffering from disorders of this nature are usually drawn from the middle walk of life, and, by reason of the pressure of household duties or the performance of the daily tasks incidental to their vocation, are entirely unable, in the slightest degree, to assist, by proper rest or procedure, the action of the administered remedy.

Many of these patients, too, suffer in silence for months, and even when forced by the extremity of their sufferings to the physician, shrink from relating a complete history of their condition and absolutely refuse to submit to an examination. Authoritative medical teaching and experience unite in forcing upon the attendant a most pessimistic view of his efforts in behalf of these sufferers under such conditions.

It is in this class of practice, where almost everything depends upon the remedy alone, that a peculiarly aggravating condition of affairs exists. A very limited list of remedies of demonstrated value is presented for selection, and I believe I am not wide of the mark in saying that, in the hands of most practitioners no remedy or combination of remedies hitherto in general use has been productive of anything but disappointment.

Some time ago my attention was drawn to Ergoapiol (Smith) as a combination of value in the treatment of a great variety of uterine disorders. Its exhibition in several cases in my hands yielded such happy results that I have used it repeatedly in a considerable variety of conditions, and with such uniformly good results that I am confirmed in the opinion that its introduction to the profession marks an era in modern therapeutics. In the treatment of irregular menstruation and attendant conditions, I have found it superior to any other emmenagogue with which I am familiar, in the following particulars:

1. It is prompt and certain in its action.

2. It is not nauseating and is not rejected by delicate stomachs.
3. It is absolutely innocuous.
4. It occasions no unpleasant after-effects.
5. It is convenient to dispense and administer.

The following clinical notes will afford a general idea of its action in a variety of cases:

CASE 1.—Mrs. — came to me presenting the following symptoms incident to a delayed menstruation: Persistent headache of a neuralgic character; dull, aching pain in limbs and lumbar region; cramp-like pains in abdomen, and considerable nausea. The menstrual period was overdue seven days, but as yet there was no appearance of flow. Her periods had always been occasions of intense suffering, but had never before been delayed. I began the use of Ergoapiol (Smith), with some misgiving, owing to the irritable condition of the stomach. One capsule every three hours was administered without any aggravation of the gastric distress. In twenty hours a normal menstruation was well under way; the flow was slightly increased over the observed on former occasions. The pains had subsided. Ergoapiol (Smith) was administered, one capsule three times a day, during the menstrual period, which terminated in five days. The patient was instructed to return for a quantity of the remedy several days before the next menstrual period. She did so, and following directions, took one capsule three times a day for three days before expected menstruation. She subsequently reported that during the period—lasting five days—there had been practically no pain, and the amount of flow was, as far as she could judge, normal.

CASE 2.—Miss —, aged 30, had been a sufferer for years with dysmenorrhea. For about three years had suffered with leucorrhea, particularly annoying after each menstrual period. Had undergone treatment at different times for the leucorrhea and dysmenorrhea, but had never experienced permanent benefit. She had been obliged to spend the couple of days of each period in bed. She consulted me about one week before her period. Examination revealed a purulent discharge oozing from os cervix and a rather large uterus. There was no displacement. She was put upon Ergoapiol (Smith), one capsule three times a day. The onset occurred one day earlier than expected and was attended with considerable pain. The patient was, however, able to attend to her usual duties, a state of affairs such as had not been experienced for some years. At the onset of the flow Ergoapiol (Smith) was administered, one capsule every two hours. The effect was astonishing. In eight hours the pains had well-nigh subsided and there was practically no discomfort, except some pain in back.

CASE 3.—Miss —, aged 21, had suffered for two years with irregular and painful menstruation. Had commenced to menstruate when 16, menses being very scanty, but regular and accompanied with but slight degree of suffering. Was never of a very robust physique, but in the main healthy. When about 19, considerable nervous trouble was inaugurated by grieving over a great bereavement, and the menses became more and more painful. The anguish became such a horror to her that she frequently resorted to morphine, partly to allay pain and partly to procure sleep. Fortunately, she had not as yet contracted the habit, but the tendency was undoubtedly in that direction. When first consulted by her, examination was not granted. Menses appearing shortly afterward, was called upon to afford relief. Flow was very scanty and clotted. There were sleeplessness, terrific headache, pain in back, constipation, etc. Ergoapiol (Smith) was administered, one capsule every three hours. Flow was considerably increased, there was a gradual lessening of all the suffering, and almost complete relief in twelve hours. This young woman has been placed upon Ergoapiol (Smith), one capsule twice daily for one week preceding appearance of menses, and has passed through several periods with very little suffering. An examination made recently showed a marked retroversion and very sensitive cervix. A properly applied supporter will doubtless work considerable benefit in her case, but it can not be disputed that the comparatively easy menstruations occurring recently, in spite of the displacement, were due entirely to Ergoapiol.

CASE 4.—Miss —, aged 18, had always been regular in menstruating. Could get no history of any previous disorder within patient's knowledge. Contracted a heavy cold about time of menstrual epoch, and was much alarmed by non-appearance of flow. Discomfort was not marked. Ergoapiol (Smith), one capsule three times a day, was prescribed. Reported later that flow was established in twenty-four hours after treatment was commenced. The delay in this case was about four days.

CASE 5.—Mrs. — consulted me, giving the following history: Three months previously had had a profuse uterine hemorrhage, occurring about the time of menstrual period. As she had for a number of years menstruated only at intervals of about six or seven weeks, the fact that menstruation had been suspended for six weeks before the date of trouble was not especially significant. The hemorrhage, which was at no time alarming, had continued for several days. Since that time there had been an almost constant wasting and at times a considerable flow. Her condition was practically invalid. Examination revealed a gaping os, a cervix exceedingly tender and abraded and a large uterus. Before resorting to curettement it seemed advisable to try other measures. Ergoapiol (Smith), one capsule every three hours, was prescribed. In about twenty-four hours there was a decided increase in the discharge, which consisted of clots and considerable debris. There were some pains of a cramp-like nature. The discharge began to grow less in about four days and ceased entirely in one week. There was a marked improvement in general condition. Local treatment entirely removed the tenderness and abraded condition of cervix. Ergoapiol (Smith) was administered several days before next menstrual period and resulted in a very satisfactory period. In this case it appears to me the remedy saved the patient the ordeal of curettement, acting as a prompt uterine stimulant. Her condition locally and generally has since steadily improved.

